# Alcohol exposure leads to unrecoverable cardiovascular defects along with edema and motor function changes in developing zebrafish larvae

**DOI:** 10.1242/bio.019497

**Published:** 2016-07-15

**Authors:** Xu Li, Aiai Gao, Yanan Wang, Man Chen, Jun Peng, Huaying Yan, Xin Zhao, Xizeng Feng, Dongyan Chen

**Affiliations:** 1 Tianjin Key Laboratory of Tumor Microenvironment and Neurovascular Regulation, Department of Histology and Embryology, School of Medicine, Nankai University, Tianjin 300071, China; 2 State Key Laboratory of Medicinal Chemical Biology, Key Laboratory of Bioactive Materials, Ministry of Education, College of Life Science, Nankai University, Tianjin 300071, China; 3 The Institute of Robotics and Automatic Information Systems, Nankai University, Tianjin 300071, China

**Keywords:** Zebrafish, Alcohol, Heart, Blood vessel, Edema, Fetal alcohol syndrome

## Abstract

Maternal alcohol consumption during pregnancy can cause a series of developmental disorders in the fetus called FAS (fetal alcohol syndrome). In the present study we exposed zebrafish embryos to 1% and 2% alcohol and observed the morphology of heart and blood vessels during and after exposure to investigate motor function alterations, and damage and recovery to the cardiovascular system. The results showed that alcohol exposure could induce heart deformation, slower heart rate, and incomplete blood vessels and pericardium. After stopping exposure, larvae exposed to 1% alcohol could recover only in heart morphology, but larvae in 2% alcohol could not recover either morphology or function of cardiovascular system. The edema-like characteristics in the 2% alcohol group became more conspicuous afterwards, with destruction in the dorsal aorta, coarctation in segmental arteries and a decrease in motor function, implying more serious unrecoverable cardiovascular defects in the 2% group. The damaged blood vessels in the 2% alcohol group resulted in an alteration in permeability and a decrease of blood volume, which were the causes of edema in pathology. These findings contribute towards a better understanding of ethanol-induced cardiovascular abnormalities and co-syndrome in patients with FAS, and warns against excessive maternal alcohol consumption during pregnancy.

## Abstract (precis)

**Summary:** Alcohol exposure causes cardiovascular defects along with edema and motor function changes in zebrafish larvae that are unrecoverable in the post-exposure period.

## INTRODUCTION

FAS (fetal alcohol syndrome) is caused by excessive maternal alcohol consumption during pregnancy and leads to a series of developmental disorders of fetuses, including growth retardation, impairment of central nervous system, craniofacial defects and abnormalities of organs such as the heart (Abel, 1995; Jones, 2003). About half of the babies born with FAS suffer from various congenital heart defects (CHDs) and display ventricular septal defects, atrial septal defects as well as Fallots teralogy (Burd et al., 2007; Cavieres and Smith, 2000; Loser et al., 1992). The differences in malformation induced by FAS are due to various doses and timing of exposure to alcohol (Gemma et al., 2007). Many animal species have been employed to explore the effect of alcohol on heart development, such as chick, rodents and zebrafish (Bruyere and Stith, 1994; Cavieres and Smith, 2000; Daft et al., 1986; Dlugos and Rabin, 2010; Sarmah and Marrs, 2013). In chick, ventricular septal defect (VSD), double outlet right ventricle and defects in aortic arch and subclavian artery were caused in chick embryos by acute alcohol exposure for 72-80 h (Fang et al., 1987). Bruyere and Stith (1993) studied the effect of alcohol on the hearts of three district commercial strains of White Leghorn chick embryos, and found that the different strains showed different susceptibilities to the induction of VSD by ethanol (Bruyere and Stith, 1993). The defects in embryonic cardiac blood flow during cardiogenesis was associated with the ethanol-induced intracardiac defects in chick embryos (Bruyere and Stith, 1994). Another study also showed that genetic background strongly regulates the effects of alcohol on heart development (Cavieres and Smith, 2000). In mice, alcohol exposure on gestation day (GD)8, 9 and 10 by intraperitoneal injection or gavage could induce congenital heart defects with malformation of ventricular septal defects (Webster et al., 1984) and acute exposure to alcohol could induce abnormal heart and great vessel development (Daft et al., 1986).

The zebrafish (*Danio rerio*) has become a promising animal model for developmental biology, pharmacology and genetics because of unique advantages such as the strong similarity of its genome to the human genome, rapid development *in vitro* with well characterized developmental stages, easily observed transparent embryos, high fecundity, low-cost husbandry and housing (Westerfield, 2007). Zebrafish have also been widely used in exploring the effect of alcohol on development (Bilotta et al., 2004; Dlugos and Rabin, 2010; Joya et al., 2014). Alcohol-exposed zebrafish embryos displayed axial malformations, delayed development, epiboly and gastrulation defects, abnormal notochord and spinal cord, and smaller eyes (Arenzana et al., 2006; Laale, 1971; Zhang et al., 2010). The severity of these effects was related with the amount of ethanol and the timing of ethanol exposure. Blader and Strahle (1998) reported that alcohol exposure could impair the migration of the prechordal plate mesoderm and result in the loss of expression of genes important in brain development. Ethanol treatment also caused defects in learning and memory, increased cell death in the CNS, skeletal dysmorphogenesis, and alterations in startle reflex responses (Carvan et al., 2004). Studies have found that alcohol could alter heart volumes, reduce the thickness of ventricular wall, and lower heart rate (Dlugos and Rabin, 2010). Sarmah and Marrs (2013) found that exposure during the entire period of cardiogenesis could cause severe defects in heart structures. Folic acid was effective in rescuing defects in heart development induced by ethanol (Sarmah and Marrs, 2013). However, whether those defects in the cardiovascular system and body morphology, and motor function alterations could recover after stopping exposure remains unknown.

Here, we exposed transgenic zebrafish Tg[cmlc2: eGFP] and Tg[fli1:eGFP] embryos to alcohol to investigate the effect of alcohol on development of the cardiovascular system, particularly in the recovery ability of the defective heart, body morphology and alterations to motor function after exposure. The results showed that alcohol exposure could induce cardiovascular defects such as deformed heart, slower heart rate, incomplete blood vessels, pericardium edema and so on. After stopping exposure, larvae exposed to the lower concentration (1%) could recover only in heart morphology, but larvae exposed to the higher concentration (2%) could not recover in heart morphology. The defects in heart rate and blood vessels, and the edema-like characteristics of larvae exposed to the higher concentration became more conspicuous afterwards. Histological examination confirmed that edema in muscle tissue in larvae treated with 2% alcohol, and confocal imaging showed drastic destruction of dorsal aorta and coarctation in segmental arteries in the 2% group. The damaged blood vessels found in the 2% group resulted in increased permeability and decreased blood volume, which allowed fluid enter the interstitium spaces. Furthermore, motor function decreased by alcohol could not be recovered in the phase after exposure. The results indicate that the severe impacts of alcohol exposure on the cardiovascular system could lead to edema and further motor function changes instead of recovering in the period of post-alcohol exposure. These findings provide a reference for better understanding of ethanol-induced cardiovascular abnormalities and co-syndrome in patients with FAS, and play a warning role regarding excessive maternal alcohol consumption during pregnancy.

## RESULTS

### Defects in the cardiovascular system fail to recover completely following alcohol exposure

**Fig. 1.**
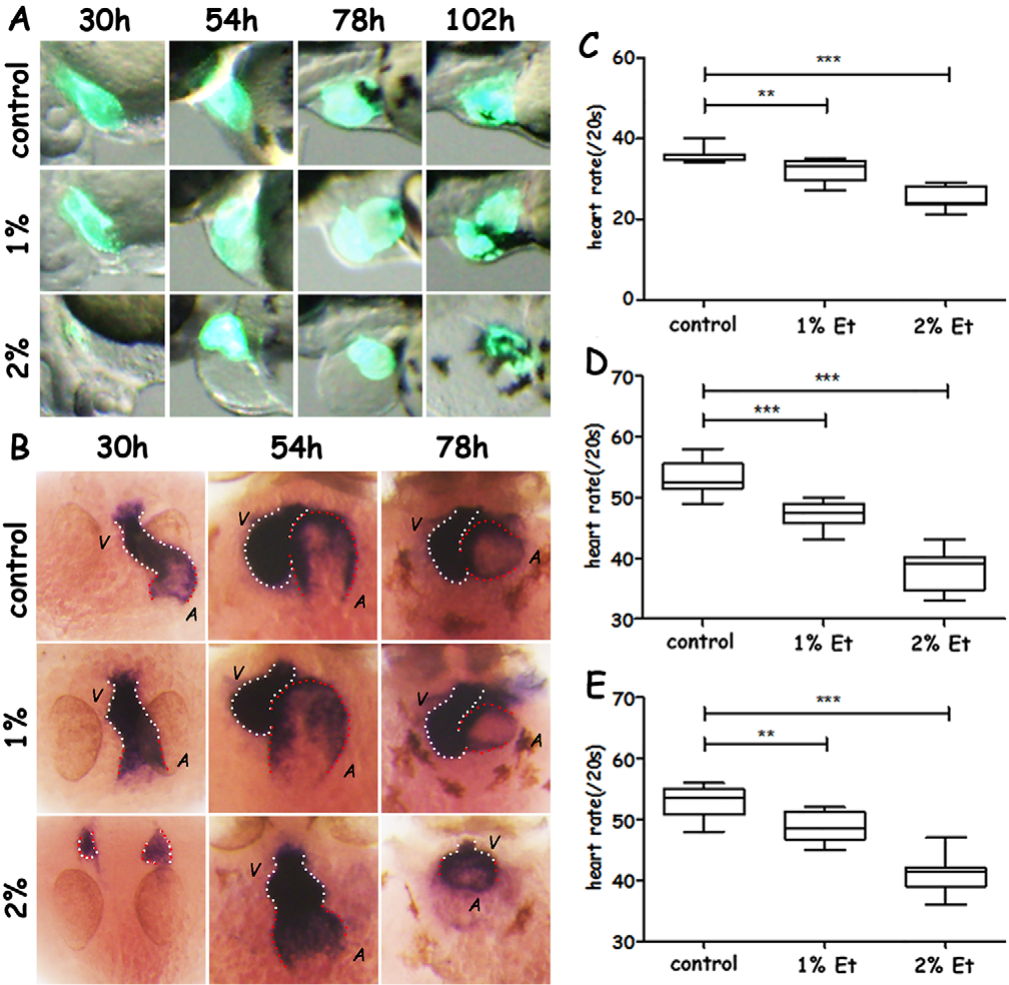
**Phenotypic analysis, expression of *cmlc2* and heart rate analysis after early exposure of alcohol.** (A) Morphological characteristics of the heart in alcohol-exposed larvae. (B) Ventral view of the expression of *cmlc2* in the heart of alcohol-treated larvae with the head at the top at 30, 54 and 78 hpf. (C-E) Heart-rate analysis in ethanol-treated larvae at 30 hpf (C), 54 hpf (D) and 78 hpf (E). Red dotted line, atrium; white dotted line, ventricle. Error bars represent mean±s.e.m.; **P<0.01, ***P<0.001 by one-way ANOVA; *n*=48 for each group.

In order to investigate the recovery condition of cardiovascular system after alcohol exposure, the morphology of heart was observed under fluorescence stereomicroscope from 30 hours post-fertilization (hpf) to 102 hpf at 24-h intervals after treatment with alcohol at concentrations of 1% and 2% from 10 hpf to 30 hpf. No obvious difference in heart morphology was found between the control group and the 1% alcohol-treated group after 30 hpf (Fig. 1A). Compared with the control or 1% alcohol-treated group, the hearts of the 2% alcohol group were much smaller, with edema-like increscent pericardium at 30 hpf (Fig. 1A). As development continued, the hearts in the 2% group became bigger and the pericardium edema became more severe. Thus, the hearts of the 2% group could not recover in the period post-alcohol exposure (from 30 hpf to 102 hpf).

To further explore the heart morphology changes after exposure to alcohol, the expression of *cardiac myosin light chain 2* (*cmlc2*), *atrial myosin heavy chain* (*amhc*) and *ventricular myosin heavy chain* (*vmhc*) were investigated in the ethanol-treated and control larvae. In the control group, expression of *cmlc2*, *amhc* and *vmhc* indicated the morphology of whole heart, atrium and ventricle, respectively. At 30 hpf, when the treatment with alcohol finished, the expression patterns of *cmlc2*, *vmhc* and *amhc* showed that the heart in control larvae was composed of a single atrium and ventricle, with ventricle along the midline and atrium on the lower left (Fig. 1B; Fig. S1). In the 1% alcohol-treated group, the structure of the heart with a single atrium and ventricle was also found, but the ventricle and atrium were both along the midline (Fig. 1B; Fig. S1). No typical heart structure was found in the 2% ethanol-treated larvae at 30 hpf; a normal atrium and ventricle were not formed and two cardiac progenitor cell populations were situated on both sides of the midline (Fig. 1B; Fig. S1). At 54 hpf and 78 hpf, the morphology of the hearts in 1% ethanol-treated larvae was similar to that of the control group, with the ventricle on the right and atrium on the left, which suggested that the heart morphology could self-restore (Fig. 1B; Figs S2, S3). However, in the 2% alcohol-treated group at 54 hpf, a single atrium and ventricle were formed, but the atrium and ventricle were along the midline (Fig. 1B; Fig. S2). At 78 hpf, the cardiac chambers were much smaller in 2% ethanol treated group (Fig. 1B; Fig. S3).

In addition, heart rate was also measured to evaluate heart function. The heart rates of larvae in both ethanol-treated groups were slower than untreated larvae at 30 hpf, and were still not normal at 54 and 78 hpf. The heart rates of 2% ethanol-treated larvae decreased much more than 1% ethanol-treated larvae at all the time points recorded (Fig. 1C-E).

**Fig. 2.**
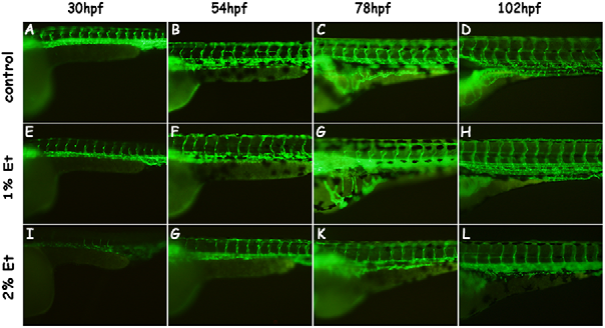
**The effect of alcohol exposure on the blood vessels of zebrafish larvae at 30, 54, 78 and 102 hpf.** (A-D) Morphology of blood vessels in control group at 30, 54, 78 and 102 hpf. (E-H) Morphology of blood vessels in 1% group at 30, 54, 78 and 102 hpf. (I-L) Morphology of blood vessels in 2% group at 30, 54, 78 and 102 hpf.

The effect of alcohol on the blood vessels was explored using the Tg (fli1: eGFP) zebrafish embryos. In the 1% alcohol-treated group at 30 hpf, the blood vessels, such as segmental arteries and dorsal aorta, were narrow compared with the control group (Fig. 2). In the 2% alcohol-treated groups, the number of blood vessels formed was small (Fig. 2). After stopping alcohol exposure, defects in the blood vessels in alcohol-treated larvae was mitigated gradually. At 102 hpf, no distinct defect of blood vessels could be detected in 1% alcohol treated-larvae, but in 2% alcohol-treated larvae the blood vessels were still fewer in number and narrower than larvae in the control group.

Body morphology was also observed and the head and eye area and body length were measured to quantify changes (Fig. S4). Compared with the control group, the alcohol-treated embryos showed shorter body length, smaller head and eyes at 30 hpf, after alcohol exposure was finished (Fig. 1B-D). The abnormal phenotype was more severe in 2% alcohol-treated embryos than in 1% alcohol-treated embryos (Fig. S4A). After culture in system water, the malformation in 1% alcohol-treated larvae recovered gradually, but malformation in 2% alcohol-treated larvae could not recover, in the same way as the typical ‘edema-like-change’ (Fig. S4A). The defects in body length in 2% alcohol-treated larvae became more severe as development progressed (Fig. S4A).

### Higher alcohol exposure causes edema, which is related to severely damaged blood vessels in the post-exposure phase

**Fig. 3.**
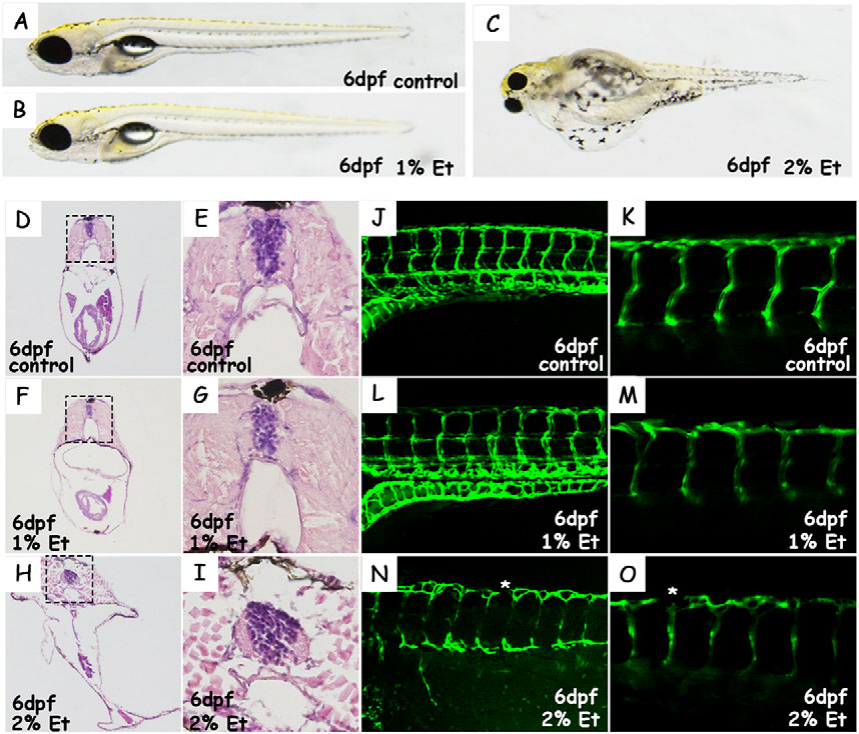
**Morphology, histopathological examination and vascular structure in ethanol**-**treated and untreated larvae.** (A-C) Morphological characteristics of ethanol-treated and untreated larvae at 6 dpf. (D-I) H&E staining of transverse sections at site of abdomen in untreated and ethanol-treated larvae. Boxed areas in D,F,H magnified in E,G,I. (J,L,N) Reconstructed images of vascular structure in untreated and ethanol-treated larvae by confocal imaging. (K,M,O) Tomography of vascular structure in control and alcohol-treated larvae by confocal imaging. Asterisk in N,O indicates destruction in dorsal aorta.

At 6 dpf no significant morphological differences were observed between untreated and 1% alcohol-treated larvae, while the typical edema-like characteristic was conspicuous in the 2% ethanol-treated group (Fig. 3A-C). As these larvae grew, the pericardium edema became more and more severe when the larvae were cultured in egg water. At 6 dpf the edema in larvae treated with 2% alcohol was drastic, with a large transparent bubble on each side of the body. In order to detect the characteristic of typical edema-like phenotype found in the 2% alcohol-treated group, histopathological examination was performed by H&E staining (Fig. 3D-I). No obvious difference was found between control and 1% alcohol-treated larvae; however, in the sections from larvae in the 2% alcohol-treated group, enlarged intramuscular space and isolated muscle bundles were found in muscle tissue.

Additionally, a confocal microscope was employed to investigate the complex effect of alcohol on the blood vessels, whose structure showed a high correlation with the edema-like phenotype found in the 2% alcohol-treated group (Fig. 3J-O). These results showed that the blood vessels in alcohol-treated larvae were destroyed to different degrees under different alcohol concentrations. In the 2% alcohol-treated group, much more conspicuous destruction in dorsal aorta, and clear coarctation in segmental arteries, was observed, which resulted in alterations in permeability and a decrease of blood volume. These alterations in permeability and decreased blood volume could lead to these typical edema-like changes.

### Changed motor function induced by alcohol exposure could not be recovered at 6 dpf

**Fig. 4.**
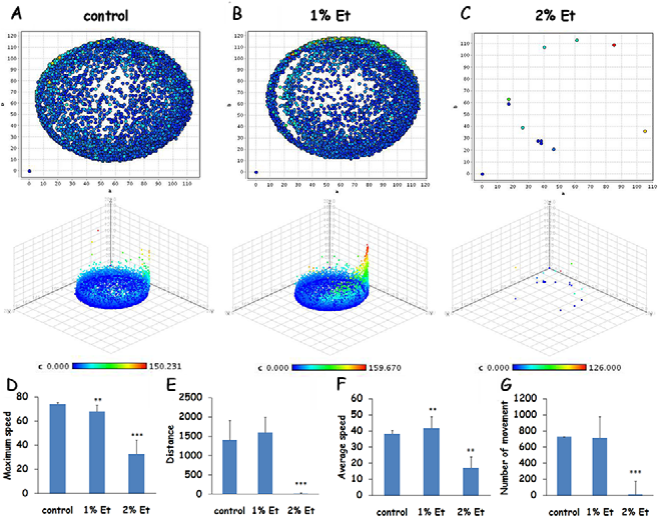
**Motor function changes at 6 dpf are induced by early exposure to alcohol.** (A-C) Representative 2D and 3D reconstructions of movement trajectory for untreated larvae (A), 1% alcohol-treated larvae (B) and 2% alcohol-treated larvae (C) at 6 dpf. Different colors indicate different speeds; blue represents low speed, and red represents high speed. The *z* axis indicated time, and 3D reconstructions revealed the spatiotemporal behavioral phenotypes of larvae. (D-G) Bar graphs depicted the four behavioral parameters; maximum speed, total distance, average speed and the number of movements, for control and 1% and 2% ethanol-treated groups. Error bars represent mean±s.e.m.; **P<0.01,***P<0.001 by one-way ANOVA; *n*=48 for each group.

To further explore the motor function abnormality caused by the edema-like changes, the treated larvae were cultured in system water for a behavioral test. Similar to the control group, the larvae treated with 1% ethanol were hyperactive and possessed of thigmotaxis. They constantly swam along the wall of a 48-well plate, and sometimes also crossed the middle of the well (Fig. 4A-C). For maximum speed, total distance, average speed and the number of movements, there were no obvious differences between control and 1% ethanol-treated groups (Fig. 4D-G). It was worth noting that treating with 1% ethanol induced increased speed as time went by. The larvae exposed to 2% ethanol displayed very little locomotor activity, probably because of morphological changes. From the figure of 3D reconstructions ( Fig. 4C), we found the larvae almost kept immobile. Treatment with 2% ethanol also resulted in a low value for total distance and the number of movements. Thus, in the post-exposure phase the decreased motor function of larvae exposed to 2% alcohol is not recovered.

## DISCUSSION

It has been reported that alcohol exposure can affect cell movement during gastrulation, induce split axes, disrupt extraembryonic microtubule cytoskeleton and embryonic blastomere cell adhesion, produce epiboly and gastrulation defects (Blader and Strahle, 1998; Carvan et al., 2004; Zhang et al., 2010). In zebrafish, cardiac progenitors form in ventral and lateral regions of the embryo at 5 hpf. In our study, in order to exclude the extensive effect of alcohol on cell movement during gastrulation, we exposed the embryos to alcohol from 10 hpf when gastrulation finished. Alcohol exposure could induce smaller head and eyes, shorter body, defects in heart morphology and function, as well as changes in motor function, which were in accordance with the phenotypes of FAS. Here, we examined the recovery of heart, blood vessels and motor function after the phase of alcohol treatment. Alcohol (1% and 2%) can induce defects in heart, blood vessels and motor function, which may or may not be recoverable in the phase after the alcohol exposure.

The higher concentration of alcohol (2%) caused a more severe impact, preventing the myocardial precursors from fusing and forming the heart cone at 30 hpf. Although the hearts of larvae treated with 2% alcohol can form the heart tube after alcohol exposure, the morphology of the heart was much smaller and the heart rates were much slower than untreated hearts at 54 hpf, 78 hpf and 102 hpf, which suggesting that the higher concentration of alcohol exposure (2%) could cause unrecoverable defects in heart formation and function. Compared with hearts in the 2% alcohol treatment group, hearts treated with 1% alcohol can recover to normal morphology following 24 hours' culture in egg water after ethanol exposure stopped at 30 hpf, but the heart rates were still a little slower at 54 hpf and 78 hpf, which shows a defect in function after the phase of alcohol treatment. Thus, the damage caused by alcohol on heart development is unrecoverable in the period after the phase of alcohol treatment.

In our study, recovery of blood vessels was also detected in the phase after the alcohol exposure. Destroyed blood vessels were found in both groups, particularly in the 2% group with damaged dorsal aorta and coarctate segmental arteries at 6 dpf. These findings implied that the defects in blood vessels caused by alcohol could not self-restore either.

Moreover, edema was found only in the 2% group in the phase after the alcohol exposure. Edema is an abnormal accumulation of body fluid in the interstitium, located in the cavities of the body and beneath the skin. Pathologically, defects in endothelial permeability can lead to edema and increase the interstitial pressure (Dejana et al., 2009). Defects in heart function are also a cause of edema. In the 2% alcohol-treated larvae, destroyed blood vessels were observed, such as damaged dorsal aorta and coarctate segmental arteries, which increased the permeability of blood vessels and allowed more fluid to enter the interstitium, thus causing edema. On the other hand, in the 2% group the defects in the heart were more serious and could also have lead to the edema phenotype. However, only pericardium edema was detected in the 1% group by phenotype observation and histological examination, which may have resulted from the slight damages caused by 1% alcohol to the blood vessels and heart. Additionally, motor function decreases caused by alcohol could not be recovered in the phase after exposure, especially in the 2% group, which may be due to the severe edema in the tissue.

In summary, whereas heart deformations induced by a lower concentration of alcohol (1%) can recover gradually, changes to heart rate and damage to blood vessels were still obvious after a long recovery time. A higher concentration (2%) of alcohol can induce more severe damage which cannot be alleviated afterwards. Incomplete blood vessels were induced by both 1% and 2% alcohol exposure, but only 2% alcohol-exposed larvae showed tissue edema resulting from a severely deformed heart as well as the increased endothelial permeability induced by serious damage to blood vessels, which enables a better understanding of ethanol-induced cardiovascular abnormalities and co-syndrome in patients with FAS. These findings also demonstrate the risks of excessive maternal alcohol consumption during pregnancy.

## MATERIALS AND METHODS

### Zebrafish husbandry

Zebrafish (AB strains; Tg[cmlc2:GFP] and Tg[fli1:EGFP] transgenic lines) were raised at 28.5°C on a 14 h light/10 h dark cycle in regular tank water (KCl 0.05 g/l, NaHCO_3_ 0.025 g/l, NaCl 3.5 g/l and CaCl_2_ 0.1 g/l, with 1 mg/l methylene blue, pH 7.0). All the experimental protocols and zebrafish procedures were approved by the Committee for Animal Experimentation of the College of Life Science at Nankai University (no. 2008) and were performed in accordance with the NIH Guide for the Care and Use of Laboratory Animals (no. 8023, revised in 1996).

### Alcohol exposure

Fertilized eggs were collected and cultured at 28.5°C in regular tank water. Embryos at 10 hpf were selected and divided into three groups, then exposed to system water (control), or 1% and 2% alcohol in regular tank water. At 30 hpf, the three groups of embryos were transferred to regular tank water and cultured again at 28.5°C for 6 days (Fig. S5).

### Morphological observation

Zebrafish embryos in the alcohol-exposed groups were observed under a stereo microscope (Olympus ZX10, Japan), and photographed at 30, 54, 78, 102 and 120 hpf for morphological analysis. Meanwhile, head area, eye area and body length of larvae were measured by Olympus BP2-BSW software. More elaborate images of blood vessels were obtained using an Olympus Fluoview FV1000 confocal microscope. 3D reconstructions were analyzed using FV10-ASW 3.1 software (Olympus).

### Whole-mount *in situ* hybridization

The embryos from each group were collected at 30, 54, 78 and 102 hpf and fixed overnight in 4% paraformaldehyde solution made with phosphate-buffered saline at 4°C. Digoxigenin-labeled probes (for *cmlc2*, *amhc* and *vmhc*) and whole-mount *in situ* hybridization were performed as previously described (Wang et al., 2015).

### Histopathological examination

At 6 dpf, zebrafish larvae were fixed in 4% paraformaldehyde overnight and then embedded in paraffin. 8-µm-thick sections were cut and stained with haematoxylin and eosin. The sections were evaluated and photographed using an Olympus BX51 light microscope equipped with an Olympus CCD DP71 camera.

### Behavioral test

We utilized a custom video-tracking system to record the locomotor activity of zebrafish larvae exposed to 1% and 2% ethanol for 30 min. In the system, all the equipment was fixed in a sealed box with constant temperature, humidity and light cycle. A 48-well plate was placed on a transparent acrylic shelf to hold the larvae. An infrared LED array was utilized as a back light source. A camera (MV-VS078FM, Microvision), fitted with a fixed-angle megapixel lens (MP5018, Computar) and an infrared filter, was used for vertical observation of the larvae. The system captured high-quality images at 30 frames per second with a resolution of 1024×768. An algorithm based on the subtraction of the adjacent frame was applied. To suppress background noises, all the images were processed through the Gaussian filter. The minimum bounding rectangle (MBR) of the largest connected component was calculated and the center of the MBR was referred to as the location of the larva. If the largest connected component of the MBR was greater than threshold area, which was set to approximately 75% of the body area of the larva, then the larva was considered mobile; otherwise the larva was considered immobile. Statistical differences in MBRs were determined by ANOVA.

Four behavioral parameters were analyzed, including the maximum speed, total distance, the average speed and the number of movements. In each experiment, 16 larvae were tested for each group, and standard system water was used for control. Error bars indicate s.e.m. in three independent measurements. *P*<0.01 (**) and *P*<0.001 (***) were set for significant differences, which were calculated via one-way ANOVA with SPSS 19.0 (IBM). In addition, we reconstructed the 2D and 3D movement trails for control and ethanol-treated groups. In reality, they were not actual 3D trajectory reconstructions in space, because the *z* axis represented time. The *x* and *y* axes indicated the horizontal and vertical ordinates, respectively, of the larva in the 48-well plate.
